# Frequency of Internet Use and Cancer Information Seeking Among U.S. Adults: A Nationally Representative Analysis of the 2024 Health Information National Trends Survey (HINTS-7)

**DOI:** 10.3390/healthcare14203437

**Published:** 2026-10-14

**Authors:** Jonathan N. Odumegwu, Emmanuel Peprah, Jackson Barth, Melody S. Goodman, Kimberly A. Kaphingst

**Affiliations:** 1 Department of Statistical Science, Baylor University, Waco, TX 76798, USA; jackson_barth@baylor.edu; 2 Implementing Sustainable Evidence-Based Interventions Through Engagement (ISEE) Lab, School of Global Public Health, New York University, New York, NY 10003, USA; ep91@nyu.edu; 3 Department of Global and Environmental Health, School of Global Public Health, New York University, New York, NY 10003, USA; 4 Department of Biostatistics, School of Global Public Health, New York University, New York, NY 10003, USA; melody.goodman@nyu.edu; 5 Huntsman Cancer Institute, University of Utah, Salt Lake City, UT 84112, USA; kim.kaphingst@hci.utah.edu; 6 Department of Communication, University of Utah, Salt Lake City, UT 84112, USA

**Keywords:** information seeking behavior, internet-use frequency, healthcare utilization, cancer survivors, health literacy, trust

## Abstract

**Background:** Rising cancer diagnoses and survivorship have increased demand for reliable cancer information. Online platforms offer resources that can strengthen health information and engagement; however, variable information quality, misinformation, and unequal digital access remain concerns. Little is known about how internet-use frequency, device type, and cancer history associate with cancer information seeking. **Objective:** To examine how internet-use frequency, device type, and personal cancer history associate with cancer information seeking. **Methods:** This study uses nationally representative data from 7245 respondents, weighted to represent approximately a sample of 261,682,768 US adults in the 2024 Health Information National Trends Survey 7 (HINTS 7). Weighted adjusted logistic regression was used to examine associations between cancer information seeking, internet-use frequency, and personal cancer history. **Results:** The odds of seeking cancer information are statistically significantly higher among those who use the internet more than once per day (adjusted odds ratio [aOR] = 2.45, 95% CI: 1.12–5.36). Individuals with a personal history of cancer also have significantly higher odds of seeking cancer information (aOR = 3.75; 95% CI: 1.23–11.5) than those with no cancer history. However, personal cancer history does not modify the relationship between internet-use frequency and seeking cancer information, suggesting that frequent daily internet exposure among US adults may independently affect the odds of seeking cancer information, regardless of personal cancer history. The proportion of participants seeking cancer information also increases significantly (*p* < 0.001) with educational attainment, from 24.8% among those with, at most, an 11th-grade education to 64.6% among college graduates. The odds of seeking cancer information were statistically significantly higher for college graduates (aOR = 3.06; 95% CI: 1.66–5.61), compared with those with ≤11 grades. **Conclusions:** Frequent internet use was associated with cancer information seeking, and there was no statistically detectable difference across personal cancer history. Public health efforts should prioritize improving digital health literacy, expanding access to credible online resources, and using culturally tailored strategies to reach populations less likely to seek cancer information.

## 1. Introduction

Cancer remains a leading cause of morbidity and mortality in the United States. As diagnoses and survivorship increase, the need for accessible, timely, and reliable information at every stage of the disease also grows [1,2]. Credible information supports prevention, informed treatment decisions, symptom management, and survivorship care [3,4,5]. Advances in early detection, treatment, and supportive care have substantially improved survival, resulting in an expanding population of individuals living with and beyond cancer that need information on supportive care and prevention whenever possible [6].

Cancer information seeking is shaped by knowledge, beliefs, perceived risk, prior experiences, and motivation to manage one’s health [5,7,8]. Consistent with established health behavior theories, including the Health Belief Model, greater perceived susceptibility and disease severity motivate health-protective action. For cancer survivors, fear of cancer recurrence—worry that cancer will return or progress—can heighten perceived threat and prompt information seeking as a way to cope and regain a sense of control [9]. This health behavior mechanism, as noted in information-seeking frameworks such as the Comprehensive Model of Information Seeking, further underscores the roles of personal experience with disease and the perceived utility of information channels [10] where the perceived benefits of health information, such as lifestyle modification, outweigh the risks [9]. Organizations, such as the NCI, the American Cancer Society (ACS), CancerCare, and Triage Cancer, have played central roles in disseminating evidence-based cancer information through public awareness campaigns and digital platforms [11,12].

The internet has reshaped how people seek health information. Many patients and caregivers search online for cancer-related guidance, from symptoms and treatment to survivorship and support [13,14,15]. Access to this information can help them participate in care decisions, communicate with providers, and find support. Several factors drive growing reliance on internet-based health information. Convenience, accessibility, anonymity, and immediacy lead an estimated 60–80% of internet users to seek health information online [16,17]. Online platforms also connect users with peer support, firsthand experiences, and practical guidance that may be difficult to obtain during clinical visits [18,19,20]. These resources can strengthen patient empowerment and health literacy, support informed decision-making, and potentially reduce unnecessary healthcare use [21,22]. In cancer care, health literacy—the ability to find, understand, and appropriately use health information—shapes how people search for, process, and act on cancer-related information [23,24]. Reasons for seeking cancer information vary significantly across life stages. Young patients and cancer survivors in early adulthood are more likely to seek specific information online about their diagnoses, treatment options, and long-term management or late treatment complications that require proactive, self-directed monitoring [25] The literature has mainly focused on communication strategies to improve cancer literacy, including simplifying language, confirming comprehension, and training healthcare providers to tailor information to diverse patient needs [26,27].

Despite these advantages, online health information varies in quality and credibility, making it difficult to distinguish evidence-based guidance from misinformation or promotional content [12,28]. Misleading information can fuel anxiety, self-diagnosis, inappropriate treatment choices, and conflict with providers [29,30,31] Consequently, understanding how people use digital cancer information is a public health priority.

Patterns of cancer information seeking can depend on how individuals access and use digital technologies. Internet-use frequency, device type, healthcare experiences, and personal cancer history may each shape information-seeking behaviors [32,33,34]. Computers often support in-depth searches, while smartphones are commonly used for quick queries, social media, and communication [35] Individuals living with cancer, survivors, and caregivers may seek online information about treatment options, symptom management, recurrence prevention, and supportive care. Survivors experiencing persistent effects such as neuropathy, fatigue, nausea, or chronic pain may be especially likely to seek guidance to manage symptoms and improve quality of life [36,37]. In addition, trust in healthcare providers also shapes how patients seek and use cancer information. Strong trust can encourage open communication, help patients ask questions and understand clinical guidance, and support their health literacy, treatment adherence, and continuity of care [38,39,40,41]. A patient’s trust in their providers correlates with their cancer-information-seeking behaviors [38]. Unfortunately, when trust is lacking, patients often avoid seeking health information (e.g., cancer information) from their providers, delay visits, avoid preventive services, not adhere to treatment, or look for health information from other sources, such as the internet [42].

Although previous studies have examined general health information seeking and internet use independently [17,43], relatively few have comprehensively evaluated how multiple dimensions of internet exposure—including frequency of internet use and the devices used to access information—are associated with cancer information-seeking behavior at the population level. Furthermore, whether these relationships differ between individuals with and without a personal history of cancer remains insufficiently understood. Addressing these gaps is important for developing effective digital communication strategies and tailoring public health interventions to populations with varying informational needs.

Using nationally representative data from HINTS 7, the present study aimed to: (1) examine the association between frequency of internet use and cancer information-seeking; (2) determine whether this association varies by personal history of cancer; and (3) investigate whether different forms of device use are associated with cancer information-seeking behavior. We hypothesized that more frequent internet use would be associated with higher odds of seeking cancer information and that this association would be stronger among individuals with a history of cancer, who may have greater informational needs related to treatment, symptom management, survivorship, and prevention of recurrence.

## 2. Methods

**Data source**: This cross-sectional study analyzed publicly available HINTS 7 data. HINTS is a nationally representative survey of civilian, noninstitutionalized adults aged 18 or older living in the U.S., designed to monitor how U.S. adults access, use, and communicate health- and cancer-related information; NCI has administered it approximately every 1–2 years since 2003. HINTS 7 employed a systematic sampling design for data collection and was conducted from 25 March to 16 September 2024, using a self-administered paper questionnaire with a push-to-web response option. A total of 7278 adults completed the survey, yielding a weighted response rate of 27.3 percent [44]. The sampling technique used four sampling strata, defined by crossing addresses in high-minority and low-minority areas with rural and urban geographic classifications. The goal of the strata was to increase the number of addresses in high-minority areas and rural areas in the sample [44]. The HINTS 7 survey also used a two-stage design: in the first stage, a stratified sample of addresses was selected from a database of residential addresses, and in the second stage, an adult in a sampled household was selected to complete the survey [44]. Every adult who completed the survey received a full-sample weight and 50 replicate weights; the full weight is used to calculate population and subpopulation estimates. Replicate weights were calculated using ‘delete one’ jackknife (JK1) replication methods. Returned surveys were screened for eligibility for inclusion in the final dataset for completeness and duplication (one household returning more than one questionnaire). Blank, incomplete, or duplicate surveys were excluded.

The survey package included a $2 pre-incentive, shown in the envelope window sent to all households, which was expected to increase the chance of opening the survey envelope and, consequently, the response rate. The incentive was later increased to $30 based on experiments with $10 and $20 incentives among 4800 respondents in the High Minority stratum, and it was sent to a subsample of nonrespondents (*n* = 13,055) using systematic sampling on 5 August 2024, to increase the response rate [44]. More survey details, such as eligibility criteria, additional geographical variables, the incentive experiments, weighting and variance estimation, respondent commitment experiments, and the sampling frame, are available on the HINTS 7 website [44]. HINTS 7 received human subjects approval from the Westat Institutional Review Board (Project #6632.05.73) on 2 May 2023 [45]. The survey also received a “Not Human Subjects Research” determination from the NIH Office of IRB Operations on 8 February 2024 (IRBID: IRB002042) [45]. The current study analyzed de-identified, publicly available data and does not require additional institutional review board approval.

**Figure 1:**
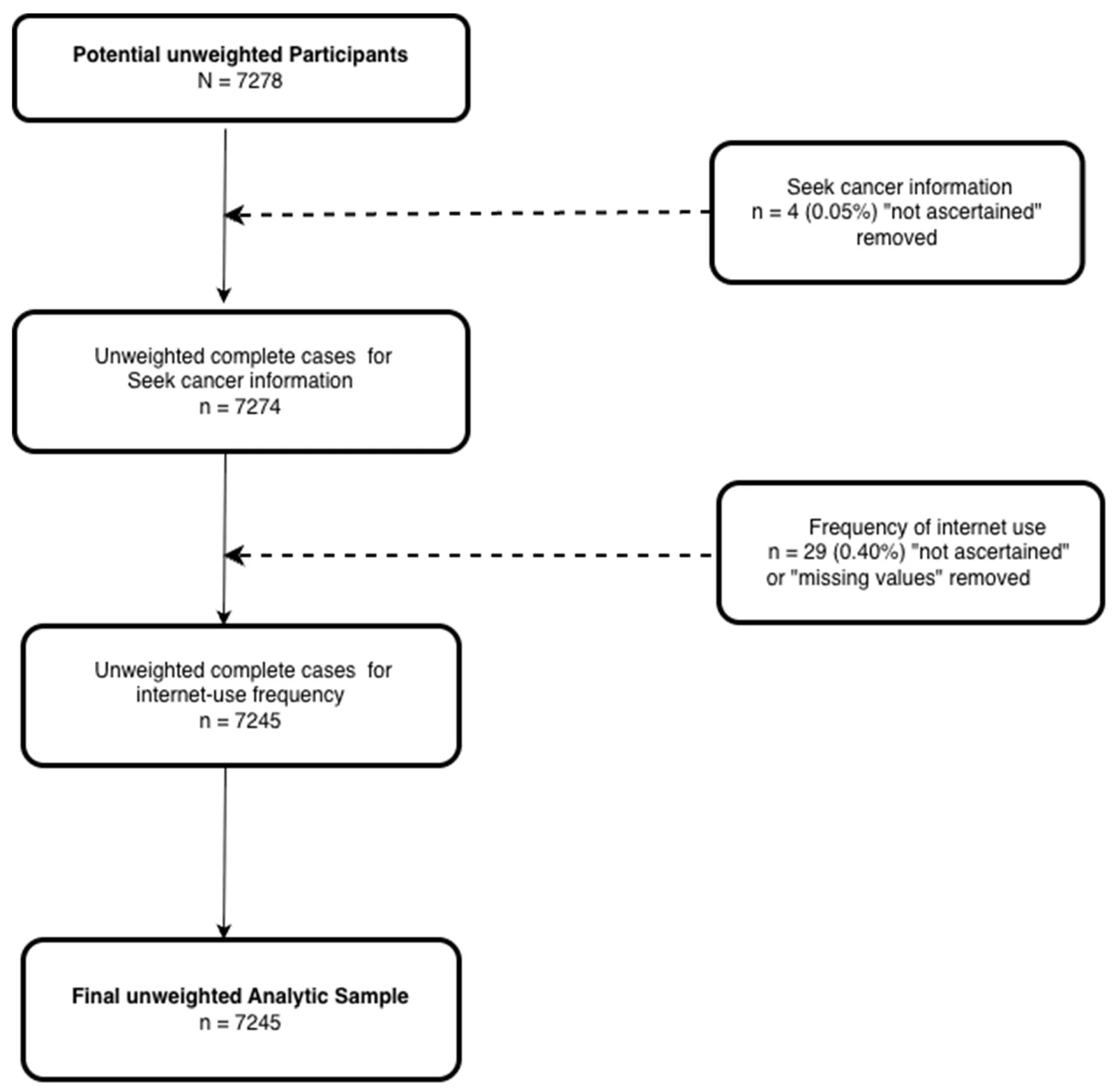
Flowchart showing how the final unweighted analytic sample was obtained.

A total of 7278 adults completed the survey, yielding a weighted response rate of 27.3 percent [44]. For the current study, we excluded participants with missing, indeterminate, or incomplete responses for primary outcome responses (seeking cancer information, *n* = 4; 0.05%) and frequency of internet use (*n* = 29; 0.40%). The final unweighted analytic sample comprised 7245 (99.6%) participants (Figure 1), yielding a weighted sample of 261,682,768.

### 2.1. Consent, Privacy, and Commitment Statements

HINTS 7 is an anonymous national survey that uses consent statements that operate in the following two ways: (1) Informed Consent Letter. The survey materials include a letter/script that informs participants about the study’s purpose, which also assures them that participation is completely voluntary, and guarantees the confidentiality of their identity [44]; (2) a commitment statement was placed at the beginning of the survey that asked the participants to be as accurate as possible and to think carefully about each question to improve data quality: “Your answers on this survey may influence cancer communication efforts and public health policy and thus affect the lives of many people in the United States. For the survey results to be most helpful, it is important that you try to be as accurate as possible and think carefully about each question. Are you willing to do this?” If the participant answers yes, the participant continues with the survey [44].

### 2.2. Measures

**Seeking cancer information**: The primary dependent variable was whether participants sought information about cancer. Participants were asked, “*Have you ever looked for information about cancer from any source?*” Response options were “Yes,” “No,” or “Not Ascertained.” Only 4 participants were coded “Not Ascertained”. We recoded not ascertained responses as missing and excluded them from further analyses.

**Frequency of internet use and device use**: The primary independent variable was the frequency of internet use. Participants were asked, “*About how often do you use the Internet, either on a computer, laptop, smartphone, or any other device?*” Response options were “More than once per day,” “About once per day,” “A few times a week,” “Less than once per week,” “Rarely,” and “Never.” We combined “less than once per week” and “rarely” and coded them as “less than once per week or rarely.” The device used to search the internet (computer, smartphone, tablet, smartwatch, etc.) was also assessed. For example, “*In the last 12 months, did you use a desktop computer or laptop*?” The responses for each were either ‘Yes’ or ‘No’.

**Healthcare utilization (Frequency of provider visits)**: The frequency of provider visits was measured by asking participants how many times they had visited their providers in the past 12 months. The single item used was: “*In the past 12 months, not counting times you went to an emergency room, how many times did you see a doctor, nurse, or other health professional to get care for yourself*?” Response options were “None,” “1 time,” “2 times,” “3 times,” “4 times,” “5–9 times,” and “10 or more times.” Based on previous studies on patients’ hospital visits, we recategorized the responses into none, low (1–2 times), moderate (3–4 times), and high/frequent (5 or more times) [44].

**Trust in doctors for cancer information:** Trust in doctors for cancer information was assessed using a single item. Participants were asked, “*In general, how much would you trust information about cancer from a doctor*?” Response options were “A lot,” “Some,” “A little,” and “Not at all.”

**Covariates**: The following sociodemographic characteristics—age, race, sex, educational attainment, personal cancer history (ever had cancer), and use of health and wellness apps—were selected as potential confounders. Age was modeled as a continuous variable in years. Sex was categorized as male, female, or do not know; race as White, Black, East Asian (Chinese, Japanese, and Korean), South Asian (for Asian Indian), Southeast Asian (for Filipino and Vietnamese), Other Asian, Multiracial, or Other (American Indian or Alaska Native and Other Pacific Islander). Educational attainment was categorized as less than or equal to (≤) 11th grade, high school/post-high school training, some college, or college graduate; cancer history was either yes or no; and any use of health and wellness apps was either yes or no. All covariates were selected a priori based on established associations with the outcome variable [36,46,47] and their theoretical relevance to frequency of internet use, the primary exposure of interest [48,49,50].

### 2.3. Statistical Analysis

To account for the HINTS 7 complex sampling design and generate nationally representative estimates, all analyses incorporated sampling weights using the Taylor series linearization approach. Weighted and unweighted descriptive statistics were calculated for the overall sample and stratified by cancer information-seeking status. Summary statistics for continuous variables are presented as weighted and unweighted medians with first and third quartiles, whereas categorical variables are summarized using weighted and unweighted frequencies and percentages. Bivariable associations were evaluated using the Kruskal–Wallis test and Pearson’s chi-squared test with the Rao–Scott adjustment.

Multivariable survey-weighted logistic regression models were employed to examine the associations between the frequency of internet use and cancer information seeking. Models were adjusted for healthcare utilization, trust in physicians, health and wellness app use, age, sex, race and ethnicity, educational attainment, and cancer history. To assess whether personal cancer history modified the association between internet use and cancer information seeking, an interaction term between internet-use frequency and personal cancer history was included in the model. Adjusted odds ratios (aORs) and 95% confidence intervals (CIs) were reported. Survey-weighted analyses were performed using the survey package in R [51], and statistical significance was assessed at *p* < 0.05. All statistical analyses were conducted using R version 4.6.1 [52].

## 3. Results

### 3.1. Participant Characteristics

Table 1 and Table S1 present weighted and unweighted descriptive statistics for the study population, respectively. Overall, approximately one-half of respondents reported having sought information about cancer (weighted: 51.3% vs. unweighted: 52.3%). The weighted median age of participants was 49 (Q1–Q3 = 34–63) years; weighted and unweighted median ages did not differ significantly between those who sought cancer information and those who did not. About 58.3% of the participants either had some college education (26.7%) or were college graduates (31.6%). Most respondents used the internet more than once per day (84.9%); 2.5% never used it. Also, about 53.9% of respondents reported utilizing healthcare (visiting their provider) moderately or frequently in the past 12 months; a total of 56% had used health and wellness apps, 84.4% reported no personal history of cancer, 82.2% reported using a computer, and 93.2% reported using a smartphone. Two-thirds of the weighted respondents were White (66.6%) and 11.1% Black; 47.4% were male, and 93.1% reported some or a lot of trust in their doctors.

### 3.2. Bivariate Analyses

**Figure 2:**
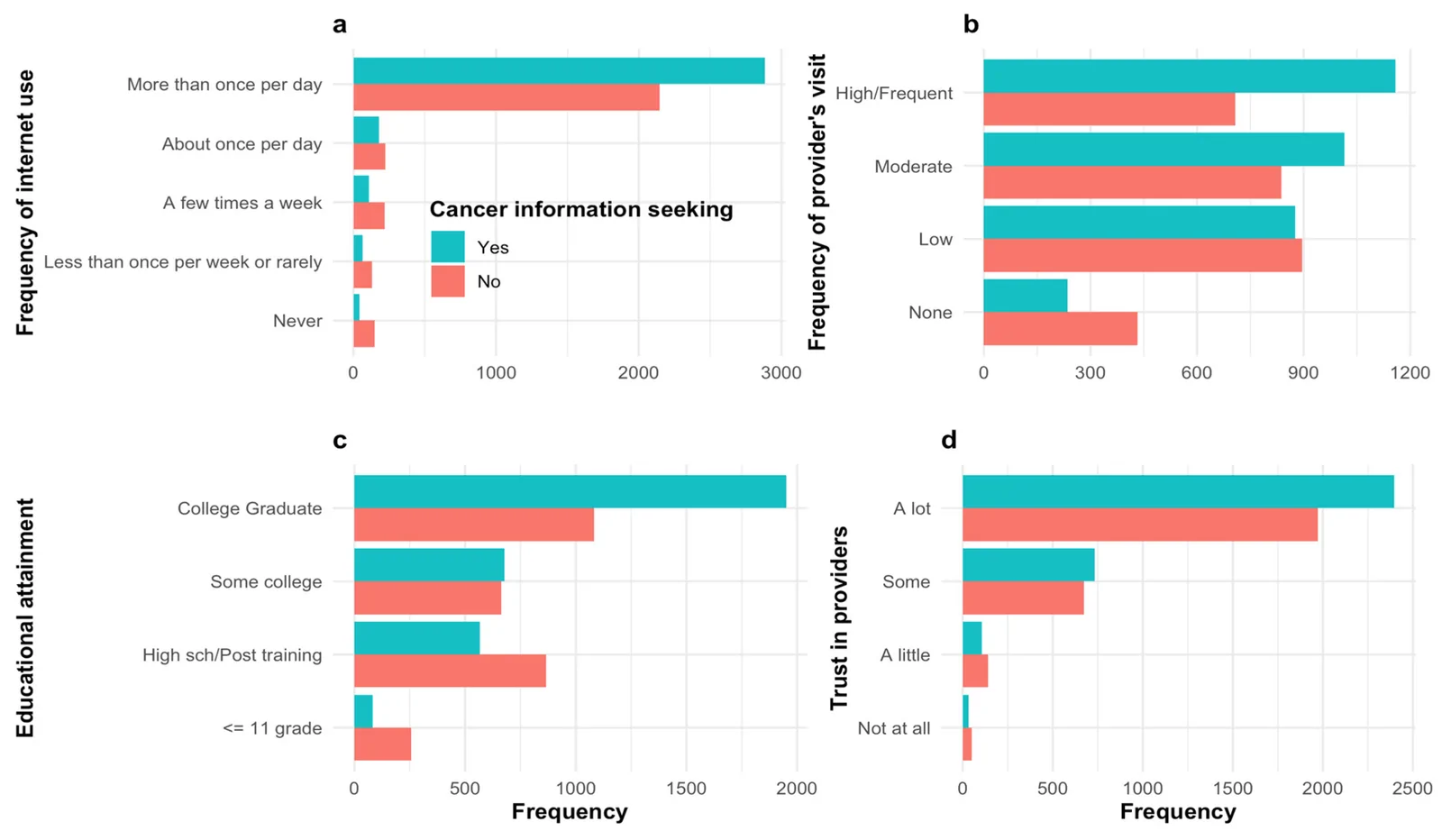
Percentages of U.S. adults who reported ever seeking cancer information, by (**a**) frequency of internet use, (**b**) number of health care visits in the past 12 months, (**c**) educational attainment, and (**d**) trust in doctors for cancer information. HINTS 7, 2024 (n = 7245).

Figure 2a–d depicts the unweighted distributions of cancer information-seeking across internet-use frequency, healthcare provider visits, trust in doctors, and educational attainment. The proportion of participants seeking cancer information increased progressively with educational attainment, ranging from 24.8% among those with ≤11th-grade education to 64.6% among college graduates (Table 1 and Figure 2c). A similar dose–response relationship was observed for internet use. Participants who reported using the internet more than once per day had the highest prevalence of cancer information seeking (53.9%), followed by those who used the internet about once per day (41.4%, Table 1; Figure 2a). In contrast, participants who never used the internet reported the lowest rate of cancer information seeking (18.8%), compared with 81.2% who reported not having sought cancer information (Table 1; Figure 2a).

Cancer information seeking was also positively associated with healthcare utilization. The proportion of participants seeking cancer information rose from 33.7% among those reporting no healthcare visits in the previous year to 60.8% among those reporting high or frequent visits (Figure 2b). Likewise, participants who used health and wellness apps were significantly more likely to seek information about cancer than non-users (59.2% vs. 41.0%; *p* < 0.001). Individuals with a personal history of cancer reported the highest prevalence of cancer information seeking, compared with those without a prior cancer diagnosis (70.9% vs. 49.1%; *p* < 0.001).

Several demographic characteristics were also associated with cancer information seeking. Significant differences were observed by race (*p* = 0.001), sex (*p* = 0.009), computer use (*p* < 0.001), and smartphone use (*p* < 0.001). White (54.2%) and South Asian (53.9%) participants reported the highest rates of seeking cancer information, followed by Multiracial participants (49.0%), and Other Asian participants with the lowest (19.9%). Female participants reported a higher rate (54.5%) of seeking cancer information than their male counterparts (48.6%) and individuals who reported that they do not know their birth sex (28.7%). Participants who used computers (55.2% vs. 31.8%; *p* < 0.001) or smartphones (52.1% vs. 37.0%; *p* < 0.001) also reported higher rates of cancer information seeking than non-device users. Trust in physicians as a source of cancer information was also significantly associated with cancer information seeking, with individuals who have some trust in physicians (46.3%) and who do not trust physicians (46.6%) reporting the lowest rates compared with their counterparts (*p* = 0.016; Table 1).

### 3.3. Multivariable Analyses

Table 2 and Table S2 present results from the survey-weighted multivariable logistic regression models. After adjustment for sociodemographic characteristics, healthcare utilization, trust in physicians, health and wellness app use, and personal cancer history, several factors remained independently associated with cancer information seeking. Using the internet more than once per day was strongly associated with seeking information about cancer. However, this association did not differ by personal cancer history. Compared with participants who never used the internet, those who used it more than once per day had significantly higher odds of seeking cancer information (aOR = 2.45; 95% CI: 1.12–5.36). Similarly, a personal history of cancer was one of the strongest predictors of cancer information seeking. Individuals with a personal history of cancer had a 275% increase in odds of seeking cancer information compared with those without a cancer history (aOR = 3.75; 95% CI: 1.23–11.50). The interaction between personal cancer history and frequency of internet use was not statistically significant, indicating that the effect of personal cancer history on cancer information seeking did not vary significantly across levels of internet-use frequency (all: *p* ≥ 0.5; Table 2).

Educational attainment was also independently associated with cancer information seeking. Compared with participants with ≤11 years of education, college graduates had a 206% increase in odds of seeking cancer information (aOR = 3.06; 95% CI: 1.66–5.61). Healthcare utilization remained a significant predictor. Compared with participants reporting no healthcare visits during the previous year, those with moderate (3–4 visits) and high or frequent (≥5 visits) healthcare utilization had significantly greater odds of seeking cancer information (moderate: aOR = 1.68; 95% CI: 1.14–2.48; high: aOR = 1.88; 95% CI: 1.25–2.82). Participants who used health and wellness apps were significantly more likely than non-users to seek information about cancer (aOR = 1.67; 95% CI: 1.34–2.07).

Significant differences were observed across demographic groups. Black participants had lower odds of seeking cancer information than White participants (aOR = 0.71; 95% CI: 0.55–0.92), whereas women had higher odds than men (aOR = 1.22; 95% CI: 1.01–1.48). Among technology-related variables, computer use remained independently associated with cancer information seeking. Participants who used a computer had 75% greater odds of seeking cancer information than those who did not (aOR = 1.75; 95% CI: 1.33–2.31). In contrast, smartphone use and trust in physicians were no longer significantly associated with cancer information seeking after adjustment for other covariates (all: *p* ≥ 0.2; Table 2).

## 4. Discussion

In this nationally representative study of U.S. adults, we found that cancer information seeking was common, with approximately one-half of participants reporting that they had searched for cancer-related information. Consistent with our primary hypothesis, more frequent internet use was independently associated with higher odds of seeking cancer information. In contrast, although we hypothesized that a personal history of cancer would strengthen the association between internet-use frequency and cancer information seeking, we found no evidence that personal cancer history modified this relationship. Higher educational attainment, frequent healthcare utilization, use of health and wellness apps, computer use, female sex, and a personal history of cancer were also independently associated with cancer information seeking.

Our findings are consistent with the internet as a channel for cancer information. Participants who reported using the internet more than once per day had more than twice the odds of seeking cancer information compared with individuals who never used the internet. These findings align with previous research showing that the internet has become the dominant source of health information because of its accessibility, convenience, and immediacy [53]. It has also been estimated that more than 60% of U.S. adults who use the internet report searching for health information online, including information about symptoms, diseases, treatments, and preventive strategies [17,43,53]. Digital platforms increasingly enable individuals to become active participants in their healthcare by improving access to educational resources, facilitating shared decision-making, and enhancing self-management of chronic conditions [43].

Despite these benefits, increasing reliance on the internet also presents important challenges. The sheer volume of online health information, coupled with variable quality and credibility, may expose individuals to misinformation and make it difficult to distinguish evidence-based recommendations from anecdotal or inaccurate information [53]. Consequently, increasing internet access alone is unlikely to improve cancer health literacy—the ability to obtain, process, and understand basic cancer-related information and services [23,24]—unless accompanied by efforts to promote digital health literacy (the ability to seek, find, and understand health information from electronic sources) [54,55] and guide individuals toward trustworthy, evidence-based sources of cancer information.

Interestingly, about one in five participants who reported never using the internet still indicated they had sought information about cancer. The outcome captures information from any source; non-internet users likely used offline sources such as clinicians, family, or print media. This finding highlights that cancer information seeking extends beyond digital platforms and may occur through healthcare providers, family members, community organizations, printed educational materials, television, and other traditional media [56,57]. Therefore, continued investment in multiple communication channels remains essential to ensure equitable dissemination of cancer information, particularly among populations with limited digital access.

Healthcare utilization emerged as another important correlate of cancer information seeking. Participants reporting moderate or frequent healthcare visits were significantly more likely to seek cancer information than those who had not visited a healthcare provider during the previous year. This finding suggests that interactions with healthcare professionals may stimulate patients’ interest in learning more about cancer, either through provider recommendations or through patients’ desire to discuss what they found online with a provider. Previous studies similarly demonstrate that routine healthcare visits increase opportunities for preventive counseling, facilitate earlier disease detection, and improve adherence to recommended screening and treatment [58,59]. Rather than replacing healthcare providers, internet-based information seeking may complement clinical care by helping patients better understand what they learn during clinical visits or prepare more informed questions for their providers [60].

Educational attainment was among the strongest correlates of cancer information seeking observed in this study. College graduates had more than three times the odds of seeking cancer information compared with individuals who had no more than an 11th-grade education. This finding aligns with extensive literature showing that higher educational attainment is associated with greater health literacy, digital literacy, and confidence in evaluating health information [33]. Individuals with higher education may also possess greater skills in identifying credible online resources and interpreting complex medical information [61]. These findings underscore the importance of designing cancer education materials that are accessible to individuals with varying levels of literacy and educational attainment.

Digital engagement beyond general internet use was also associated with cancer information seeking. Participants who reported using health and wellness apps were significantly more likely to seek cancer information, suggesting that engagement with digital health technologies may encourage broader health information-seeking behaviors. Health apps increasingly provide educational resources, symptom monitoring, medication reminders, patient-reported outcomes, and secure communication with healthcare providers, making them valuable tools for supporting cancer prevention, treatment, and survivorship [62,63]. Similarly, computer use remained independently associated with cancer information seeking after adjustment for other factors. Compared with smartphones, computers may facilitate more comprehensive information searches because of larger displays, easier navigation across multiple websites, and greater functionality for reviewing detailed medical information.

The study also observed important racial differences in cancer information seeking. Black participants had significantly lower odds of seeking cancer information than White participants; the odds of seeking cancer information for Other Asian participants were similarly low, even after adjustment for socioeconomic and healthcare-related factors. This finding is concerning because timely access to accurate cancer information is fundamental to informed decision-making, cancer prevention, screening participation, and treatment engagement. This finding echoes the racial and ethnic disparities in cancer morbidity and mortality that have been driven by systemic inequities, including unequal access to health insurance, routine screenings, and quality care [64]. Previous studies have also suggested that these disparities may reflect longstanding structural inequities, lack of trust in the healthcare system, differences in healthcare access, lower health literacy, medical mistrust, experiences of discrimination within healthcare settings, and unequal access to digital resources [24,65], which may translate into less engagement in information seeking and prevention. Although our study was not designed to identify the mechanisms underlying these disparities, the findings emphasize the need for culturally appropriate communication strategies and interventions that improve equitable access to trustworthy cancer information across diverse populations.

Consistent with previous research, individuals with a personal history of cancer were substantially more likely to seek cancer information than those without a cancer diagnosis [66,67]. In our analysis, participants with a history of cancer had more than threefold greater odds of seeking cancer information, and more than 70% reported actively searching for cancer-related information. Cancer survivors often seek information regarding treatment options, symptom management, recurrence prevention, survivorship, lifestyle modifications, and supportive care to reduce uncertainty and improve disease self-management [68,69].

Contrary to our hypothesis, personal cancer history did not statistically significantly modify the effect. This suggests that although cancer survivors seek substantially more cancer information overall, the association between internet use and seeking cancer information did not differ by personal history of cancer. One possible explanation is that cancer survivors may rely more heavily on their oncology care teams than on general internet sources when seeking disease-specific information. Given concerns about the accuracy and reliability of online cancer information, survivors may prefer to seek guidance from trusted clinicians for treatment-related decisions [70]. Psychological factors may also contribute—some survivors may limit information overload because additional information may increase and heighten fear of recurrence, thereby preferring to rely on their care teams [71,72]. Alternatively, our measure of internet-use frequency captured general internet exposure rather than cancer-specific online activities. Consequently, individuals who frequently use the internet may not necessarily use it to search for cancer-related information. Future research should use more detailed measures of digital health literacy and digital technology use—including cancer websites, patient portals, online support communities, AI-enabled tools, and social media—to better understand how internet use shapes cancer information seeking among cancer survivors. We also recommend that future studies should use content-specific survey items or natural language processing of search queries or online discussions to characterize the cancer-related content people seek.

Overall, these findings highlight the increasingly important role of digital technologies in facilitating cancer information seeking while emphasizing that significant differences remain across educational, racial, and technological domains. Public health efforts should prioritize improving digital health literacy, promoting access to credible online cancer resources, and developing culturally tailored communication strategies that reach populations less likely to seek cancer information. These efforts may support informed decision-making, increase engagement in cancer prevention and screening, and ultimately help reduce disparities across the cancer care continuum.

### Study Limitations and Strengths

Several limitations include the cross-sectional design, which prevents causal inference; self-reported data, which may be subject to recall or social desirability bias; and an overall internet-use measure that did not capture cancer-specific online activities or actual time spent online, limiting our ability to determine whether participants were actively seeking cancer information or were accidentally exposed to it online. Not measuring time spent online may distort the dose–response relationship when evaluating cancer-related health outcomes. The lack of an established precedent for internet use before seeking cancer information may also create a mismatch, and the outcome captures lifetime cancer information seeking from any source; we therefore cannot attribute information seeking to the internet, and the association may partly reflect a general information-seeking disposition. Complete-case analysis for seeking cancer information and internet-use frequency variables may also have introduced bias, although missing data were minimal (<1%). Longitudinal studies that measure whether internet use precedes cancer information seeking and whether the information was sought online should be considered. Also, despite adjusting for several demographic and healthcare-related factors, residual confounding from income, health literacy, digital literacy, or broadband access remains possible.

Despite these limitations, this study has several important strengths. First, it used data from the 2024 HINTS 7, providing a large, weighted sample of 261,682,768 (with a nationally representative sample of 7245) U.S. adults. The use of survey weights and complex sampling procedures enabled nationally representative estimates and enhanced the generalizability of the findings to the U.S. civilian, noninstitutionalized adult population. Second, the study examined several dimensions of digital engagement—including the frequency of internet, computer, and smartphone use, health and wellness app use, healthcare utilization, and trust in physicians—providing a comprehensive assessment of factors associated with cancer information seeking. Third, using standardized HINTS measures, which have been rigorously developed and widely used in national cancer surveillance research, also enhances the reliability, comparability, and public health relevance of the findings.

## 5. Conclusions

In this nationally representative U.S. study, more frequent internet use was associated with ever seeking cancer information and no statistically detectable difference in this association by personal cancer history. Efforts should prioritize strengthening digital health literacy, access to trusted online resources, and culturally tailored outreach to reduce educational, racial, and digital disparities.

## Figures and Tables

**Table 1:** Weighted descriptive statistics of the sample and across seeking cancer information status; HINTS 7 (2024).

Characteristic	Overall N = 261,682,768	Seek Cancer Info		*p*-Value ^1^
		No N = 128,079,109 (48.9)	Yes N = 133,603,659 (51.1)	
**Age, Median (Q1–Q3)**	49.0 (34.0–63.0)	48.0 (32.0–64.0)	49.0 (36.0–62.0)	0.28
**Education attainment, No. (%)**				**<0.001**
≤11 grade	15,075,178.1 (5.8)	11,338,265.4 (75.2)	3,736,912.7 (24.8)	
High sch/Post training	76,093,247.7 (29.1)	43,123,636.8 (56.7)	32,969,610.9 (43.3)	
Some college	69,763,102.9 (26.7)	35,070,547.5 (50.3)	34,692,555.4 (49.7)	
College Graduate	82,622,562.8 (31.6)	29,269,255.7 (35.4)	53,353,307.1 (64.6)	
Missing response	18,128,676.0 (6.9)	9,277,403.4 (51.2)	8,851,272.7 (48.8)	
**Frequency of internet use, No. (%)**				**<0.001**
Never	6,457,792.0 (2.5)	5,246,834.0 (81.2)	1,210,958.1 (18.8)	
Less than once per week or rarely	6,579,777.5 (2.5)	3,881,795.6 (59.0)	2,697,981.9 (41.0)	
A few times a week	11,480,572.3 (4.4)	7,789,437.5 (67.8)	3,691,134.9 (32.2)	
About once per day	14,945,532.9 (5.7)	8,763,818.4 (58.6)	6,181,714.5 (41.4)	
More than once per day	222,219,092.8 (84.9)	102,397,223.3 (46.1)	119,821,869.5 (53.9)	
**Healthcare utilization, No. (%)**				**<0.001**
None	32,542,077.3 (12.4)	21,578,073.2 (66.3)	10,964,004.1 (33.7)	
Low	85,511,854.3 (32.7)	45,180,930.0 (52.8)	40,330,924.3 (47.2)	
Moderate	74,110,908.5 (28.3)	33,445,847.4 (45.1)	40,665,061.1 (54.9)	
High/Frequent	66,882,094.3 (25.6)	26,216,443.6 (39.2)	40,665,650.7 (60.8)	
Missing response	2,635,833.2 (1.0)	1,657,814.5 (62.9)	978,018.6 (37.1)	
**Used health and wellness apps, No. (%)**				**<0.001**
No	106,295,197.3 (40.6)	62,699,334.7 (59.0)	43,595,862.6 (41.0)	
Yes	148,945,642.4 (56.9)	60,841,432.2 (40.8)	88,104,210.3 (59.2)	
Missing response	6,441,927.9 (2.5)	4,538,341.9 (70.5)	1,903,586.0 (29.5)	
**Ever had cancer, No. (%)**				**<0.001**
No	220,842,630.2 (84.4)	112,347,135.3 (50.9)	108,495,494.9 (49.1)	
Yes	23,983,472.7 (9.2)	6,988,029.5 (29.1)	16,995,443.2 (70.9)	
Missing response	16,856,664.7 (6.4)	8,743,944.0 (51.9)	8,112,720.7 (48.1)	
**Race, No. (%)**				**0.001**
White	174,247,247.6 (66.6)	79,723,059.7 (45.8)	94,524,187.9 (54.2)	
Black	29,067,176.4 (11.1)	16,878,499.6 (58.1)	12,188,676.8 (41.9)	
East Asian	6,502,893.4 (10.1)	3,573,836.2 (55.0)	2,929,057.2 (45.0)	
South Asian	3,100,572.5 (1.2)	1,429,962.1 (46.1)	1,670,610.4 (53.9)	
Southeast Asian	2,696,641.4 (1.0)	1,458,785.0 (54.1)	1,237,856.4 (45.9)	
Other Asian	1,969,893.2 (0.8)	1,578,231.2 (80.1)	391,662.0 (19.9)	
Multiracial	13,821,811.7 (5.3)	7,055,005.4 (51.0)	6,766,806.3 (49.0)	
Other	4,580,976.6 (1.8)	2,575,224.4 (56.2)	2,005,752.2 (43.8)	
Missing response	25,695,554.8 (9.8)	13,806,505.1 (53.7)	11,889,049.7 (46.3)	
**Birth sex, No. (%)**				**0.009**
Male	124,007,024.7 (47.4)	63,758,232.5 (51.4)	60,248,792.2 (48.6)	
Female	118,019,374.3 (45.1)	53,727,913.2 (45.5)	64,291,461.1 (54.5)	
Do not know	1,752,658.1 (0.67)	1,250,433.0 (71.3)	502,225.1 (28.7)	
Missing response	17,903,710.4 (6.8)	9,342,530.0 (52.2)	8,561,180.4 (47.8)	
**Use computer, No. (%)**				**<0.001**
No	45,216,655.2 (17.3)	30,843,314.1 (68.2)	14,373,341.1 (31.8)	
Yes	215,026,788.0 (82.2)	96,238,725.4 (44.8)	118,788,062.6 (55.2)	
Missing response	1,439,324.4 (0.6)	997,069.3 (69.3)	442,255.1 (30.7)	
**Use smartphone, No. (%)**				**<0.001**
No	16,029,504.2 (6.1)	10,106,200.3 (63.0)	5,923,303.9 (37.0)	
Yes	243,945,217.0 (93.2)	116,832,393.8 (47.9)	127,112,823.2 (52.1)	
Missing response	1,708,046.4 (0.7)	1,140,514.7 (66.8)	567,531.8 (33.2)	
**Trust in providers, No. (%)**				**0.016**
Not at all	4,164,829.8 (1.6)	2,225,765.4 (53.4)	1,939,064.4 (46.6)	
A little	12,073,534.7 (4.6)	5,940,978.9 (49.2)	6,132,555.8 (50.8)	
Some	58,768,081.0 (22.5)	31,537,552.6 (53.7)	27,230,528.4 (46.3)	
A lot	184,643,398.4 (70.6)	86,925,998.7 (47.1)	97,717,399.7 (52.9)	
Missing response	2,032,923.7 (0.8)	1,448,813.2 (71.3)	584,110.5 (28.7)	

^1^ Design-based Kruskal–Wallis test; Pearson’s—*χ*^2^: Rao & Scott adjustment; Missing response = Missing value; **Bold** identifies variable name or significant *p*-value.

**Table 2:** Survey-weighted multivariable logistic regression of ever seeking cancer information on frequency of internet use, personal cancer history, device use, doctor’s visits, use of health and wellness apps, and other covariates, including interaction of internet-use frequency and personal cancer history; HINTS 7 (2024).

Variable Name	aOR	95% CI	*p*-Value
**Age**	1.004	0.99–1.01	0.3
**Frequency of internet use**			
Never	—	—	
Less than once per week or rarely	1.98	0.76–5.13	0.2
A few times a week	1.22	0.54–2.79	0.6
About once per day	2.10	0.71–6.26	0.2
More than once per day	2.45	1.12–5.36	**0.03**
**Ever had cancer**			
No	—	—	
Yes	3.75	1.23–11.50	**0.02**
**Educational attainment**			
≤11 grade	—	—	
High sch/Post training	1.68	0.93–3.04	0.09
Some college	1.87	1.00–3.52	0.05
College Graduate	3.06	1.66–5.61	**<0.001**
**Healthcare utilization**			
None	—	—	
Low	1.32	0.92–1.91	0.13
Moderate	1.68	1.14–2.48	**0.009**
High/Frequent	1.88	1.25–2.82	**0.002**
**Use health and wellness apps**			
No	—	—	
Yes	1.67	1.34–2.07	**<0.001**
**Race**			
White	—	—	
Black	0.71	0.55–0.92	**0.009**
East Asian	0.80	0.33–1.93	0.6
South Asian	1.03	0.44–2.40	0.9
Southeast Asian	0.75	0.33–1.69	0.5
Other Asian	0.35	0.1–1.03	0.06
Multiracial	0.82	0.53–1.29	0.4
Other	1.12	0.57–2.23	0.7
**Birth sex**			
Male	—	—	
Female	1.22	1.01–1.48	**0.04**
Do not know	0.79	0.17–3.58	0.8
**Use computer**			
No	—	—	
Yes	1.75	1.33–2.31	**<0.001**
**Use smartphone**			
No	—	—	
Yes	1.12	0.61–2.06	0.7
**Trust in providers**			
Not at all	—	—	
A little	1.03	0.35–3.01	0.9
Some	0.58	0.23–1.44	0.2
A lot	0.67	0.26–1.70	0.4
Frequency of internet use * Ever had cancer			
Less than once per week or rarely * Yes	1.42	0.25–8.09	0.7
A few times a week * Yes	0.86	0.21–3.48	0.8
About once per day * Yes	0.76	0.19–3.07	0.7
More than once per day * Yes	0.70	0.23–2.13	0.5

Abbreviations: CI = Confidence interval, aOR = Adjusted odds ratio; **Bold** identifies variable name or significant *p*-value; * is for interaction.

## Data Availability

HINTS 7 (2024) is publicly available on the National Cancer Institute (NCI) website here.

## Supplementary Materials

The following supporting information can be downloaded at: https://www.mdpi.com/article/10.3390/healthcare14203437/s1. Table S1. Unweighted descriptive statistics of the sample and across seeking cancer information status; Data source: HINTS 7 2024. Table S2. Adjusted logistic relationships between frequency of internet use, personal cancer history, device use, doctor’s visits, and use of health and wellness apps, and other covariates, and seeking cancer information, without interactions; Data source: HINTS 7 2024.
