# Supplementary material for: Frequency of Internet Use and Cancer Information Seeking Among U.S. Adults: A Nationally Representative Analysis of the 2024 Health Information National Trends Survey (HINTS-7)
Source: Healthcare (Basel). 2026 Oct 14;14(20):3437. doi: 10.3390/healthcare14203437 (PMC13704059; doi:10.3390/healthcare14203437)
Supplement: Supplementary file 1 [file healthcare-14-03437-s001.zip › healthcare-4529564-supplementary.pdf]

Table S1. Unweighted descriptive statistics of the sample and across seeking cancer information status; Data source: HINTS 7 2024.

**Characteristic**	**Overall** N = 7,245	**Seek Cancer Info**		**p-value**[1]
		**No** N = 3,531	Yes N = 3,714	
**Age, Median (Q1 - Q3)**	58.0 (41.0 - 70.0)	59.0 (40.0 - 70.0)	58.0 (41.0 - 69.0)	0.41
**Education attainment, No. (%)**				**<0.001**
<= 11 grade	440	334.0 (75.9)	106.0 (24.1)	
High sch/Post training	1,608	994.0 (61.8)	614.0 (38.2)	
Some college	1,436	722.0 (50.3)	714.0 (49.7)	
College Graduate	3,182	1,153.0 (36.2)	2,029.0 (63.8)	
Missing response	579	328.0 (56.6)	251.0 (43.4)	
**Frequency of internet use, No. (%)**				**<0.001**
Never	319	260.0 (81.5)	59.0 (18.5)	
Less than once per week or rarely	275	181.0 (65.8)	94.0 (34.2)	
A few times a week	416	274.0 (65.9)	142.0 (34.1)	
About once per day	480	274.0 (57.1)	206.0 (42.9)	
More than once per day	5,755	2,542.0 (44.2)	3,213.0 (55.8)	
**Frequency of provider visits, No. (%)**				**<0.001**
None	823	547.0 (66.5)	276.0 (33.5)	
Low	2,123	1,109.0 (52.2)	1,014.0 (47.8)	
Moderate	2,140	1,012.0 (47.3)	1,128.0 (52.7)	
High/Frequent	2,074	811.0 (39.1)	1,263.0 (60.9)	
Missing response	85	52.0 (61.2)	33.0 (38.8)	
**Used health wellness apps, No. (%)**				**<0.001**
No	3,118	1,814.0 (58.2)	1,304.0 (41.8)	
Yes	3,845	1,524.0 (39.6)	2,321.0 (60.4)	
Missing response	282	193.0 (68.4)	89.0 (31.6)	
**Ever had cancer, No. (%)**				**<0.001**
No	5,650	2,947.0 (52.2)	2,703.0 (47.8)	
Yes	1,066	285.0 (26.7)	781.0 (73.3)	
Missing response	529	299.0 (56.5)	230.0 (43.5)	
**Race, No. (%)**				**<0.001**
White	4,517	2,000.0 (44.3)	2,517.0 (55.7)	
Black	1,129	665.0 (58.9)	464.0 (41.1)	
East Asian	153	83.0 (54.2)	70.0 (45.8)	
South Asian	80	43.0 (53.8)	37.0 (46.3)	
Southeast Asian	94	44.0 (46.8)	50.0 (53.2)	
Other Asian	45	28.0 (62.2)	17.0 (37.8)	
Multiracial	310	143.0 (46.1)	167.0 (53.9)	
Other	122	73.0 (59.8)	49.0 (40.2)	
Missing response	795	452.0 (56.9)	343.0 (43.1)	
**Birth sex, No. (%)**				**<0.001**
Male	2,640	1,358.0 (51.4)	1,282.0 (48.6)	

Characteristic	Overall<br>N = 7,245	Seek Cancer Info<br>No<br>N = 3,531	 Yes<br>N = 3,714	p-value[1]
Female	3,996	1,822.0 (45.6)	2,174.0 (54.4)	
Other	43	30.0 (69.8)	13.0 (30.2)	
Missing response	566	321.0 (56.7)	245.0 (43.3)	
**Use computer, No. (%)**				**<0.001**
No	1,491	1,023.0 (68.6)	468.0 (31.4)	
Yes	5,685	2,460.0 (43.3)	3,225.0 (56.7)	
Missing response	69	48.0 (69.6)	21.0 (30.4)	
**Use smart phone, No. (%)**				**<0.001**
No	635	428.0 (67.4)	207.0 (32.6)	
Yes	6,556	3,063.0 (46.7)	3,493.0 (53.3)	
Missing response	54	40.0 (74.1)	14.0 (25.9)	
**Trust in doctor, No. (%)**				**<0.001**
Not at all	114	72.0 (63.2)	42.0 (36.8)	
A little	334	191.0 (57.2)	143.0 (42.8)	
Some	1,702	842.0 (49.5)	860.0 (50.5)	
A lot	5,017	2,373.0 (47.3)	2,644.0 (52.7)	
Missing response	78	53.0 (67.9)	25.0 (32.1)	

[1]Wilcoxon rank sum test; Pearson's Chi-squared test

Table S2. Adjusted logistic relationships between frequency of internet use, personal cancer history, device use, doctor's visits, and use of health and wellness apps, and other covariates, and seeking cancer information, without interactions; Data source: HINTS 7 2024

Characteristic	aOR	95% CI	p-value
**Age**	1.004	.998–1.011	0.3
**Educational attainment**			
<= 11 grade	—	—	
High sch/Post training	1.69	0.93–3.05	0.08
Some college	1.88	1.00–3.55	0.05
College Graduate	3.06	1.66–5.63	**<0.001**
**Frequency of internet use**			
Never	—	—	
Less than once per week or rarely	1.99	0.90–4.40	0.09
A few times a week	1.10	0.53–2.32	0.8
About once per day	1.86	0.72–4.86	0.2
More than once per day	2.15	1.04–4.44	**0.04**
**Healthcare utilization**			
None	—	—	
Low	1.33	0.92–1.91	0.13
Moderate	1.68	1.14–2.48	**0.009**

Characteristic	aOR	95% CI	p-value
High/Frequent	1.88	1.25–2.82	**0.003**
**Use health wellness apps**			
No	—	—	
Yes	1.67	1.34–2.07	**<0.001**
**Ever had cancer**			
No	—	—	
Yes	2.83	2.11–3.80	**<0.001**
**Race**			
White	—	—	
Black	0.71	0.55–0.92	**0.009**
East Asian	0.80	0.33–1.93	0.6
South Asian	1.03	0.44–2.39	0.9
Southeast Asian	0.75	0.33–1.68	0.5
Other Asian	0.35	0.12–1.03	0.06
Multiracial	0.83	0.53–1.29	0.4
Other	1.12	0.56–2.21	0.7
**Birth sex**			
Male	—	—	
Female	1.22	1.01–1.48	**0.04**
Other	0.75	0.17–3.32	0.7
**Use computer**			
No	—	—	
Yes	1.76	1.33–2.31	**<0.001**
**Use smart phone**			
No	—	—	
Yes	1.12	0.62–2.04	0.7
**Trust in providers**			
Not at all	—	—	
A little	1.04	0.37–2.98	0.9
Some	0.58	0.24–1.43	0.2
A lot	0.67	0.27–1.69	0.4

Abbreviations: CI = Confidence Interval, aOR = Adjusted Odds Ratio
